# Discovery of HY•1: A Novel Multifunctional Skincare Ingredient With Antiaging and Skin Repair Properties

**DOI:** 10.1111/jocd.70135

**Published:** 2025-05-14

**Authors:** Xilei Duan, Zhong Lu

**Affiliations:** ^1^ Department of Dermatology Huashan Hospital, Fudan University Shanghai China

**Keywords:** antiaging products, cosmetic dermatology, skin function

## Abstract

**Objectives:**

Skin aging, influenced by intrinsic and extrinsic factors, includes photoaging caused by UV radiation, leading to ROS production and skin damage. The objective of this work is to demonstrate HY‐1, a three‐peptide conjugate shown to have antioxidation, anti‐inflammatory efficacy in vitro, and clinical benefits in anti‐wrinkle, is an efficacious ingredient for skin aging.

**Method:**

The antioxidation efficacy was studied on the ROS test, and the antiaging efficacy was studied on ex vivo human skin. In the clinical study, 49 subjects aged between 26 and 54 with wrinkle concerns were recruited and instructed to apply the investigational cream containing HY‐1 for 28 days. Wrinkle attributes were assessed by dermatologists. Instrumental measurements were also conducted.

**Results:**

In vitro studies of HY‐1 on HSF and HaCaT cells demonstrated significant efficacy in scavenging reactive oxygen species (ROS) and exerting anti‐inflammatory effects, as evidenced by decreased ROS production and reduced expression of interleukin‐6 (IL‐6). Clinical study demonstrated significant improvement in skin condition, including firmness, nourishment, and anti‐wrinkle.

**Conclusion:**

HY‐1 is a novel skincare ingredient, addressing the visible manifestations of aging and photodamage. Its multifaceted capabilities, encompassing antioxidant properties, enhancement of skin firmness, nourishment, and anti‐wrinkle effects, represent a pioneering approach with the potential to provide a skincare alternative.

**Abbreviations**
DMEM: Dulbecco's modified eagle medium
ECM: extracellular matrix
FBS: fetal bovine serum
HaCat: human immortalized keratinocytes
HPLC: high performance liquid chromatography
HSF cell: human skin fibroblast cell
IL‐6: interleukin‐6
LC–MS: liquid chromatography–mass spectrometry
LPS: lipopolysaccharide
mAChR: muscarinic acetylcholine receptor
MMPs: metalloproteinases
MW: molecular weight
ROS: reactive oxygen species
UV: ultraviolet

## 1 Introduction

The skin is a vital organ in the human body, serving as an essential barrier, as well as a conduit for conduction and excretion. Skin aging is a complex process influenced by both intrinsic and extrinsic factors [1]. Based on these influencing factors, skin aging can be primarily categorized into two types: intrinsic aging (also known as natural aging or inherent aging) and extrinsic aging, which is caused by external stimuli [2].

In extrinsic aging, ultraviolet (UV) radiation‐induced skin aging accounts for over 80% and is also referred to as photoaging of the skin. The main types of UV radiation are UVB, which primarily affects the epidermis, and UVA, which can penetrate the upper dermis and is the main culprit in photoaging. Exposure to UV radiation increases the production of reactive oxygen species (ROS), which are highly reactive chemicals that can damage cell membranes, proteins, and DNA, leading to cellular dysfunction and death, thereby promoting skin aging [3]. However, it is also known that UV radiation can lead to increased secretion of cytokines, enhanced oxidative stress responses, and increased expression of matrix metalloproteinases (MMPs). These factors contribute to the degradation of the extracellular matrix (ECM), fragmentation of collagen, and increased skin inflammation [4]. The clinical manifestations of photoaging include skin roughness, dryness, increased wrinkle formation, reduced elasticity, sagging, dilated capillaries, pigmentation, and seborrheic keratoses.

Bioactive peptides are peptide compounds composed of short chains of amino acids [5], which not only have a variety of protective functions for the skin, but also can be efficiently absorbed by the skin because of their small molecular weight and are now widely used in skincare products against skin photoaging. For instance, Argireline is capable of blocking neurotransmitter signals, which can result in more relaxed and smoother skin [6]. Palmitoyl pentapeptide‐4 can modulate fibroblasts to promote the production of extracellular matrix components [7, 8].

Snake venom peptide mimics a small amino acid sequence from Waglerin‐1, which was detected as a peptide from the venom of the Wagler's pit viper and a potent inhibitor of the muscarinic acetylcholine receptor (mAChR) [9, 10]. Thus, muscles remained relaxed, and wrinkles caused by external factors are reduced [11]. It has been successfully developed as a cosmetic peptide that can be safely applied to diminish expression lines [12].

Terpenes are predominantly synthesized by an extensive range of plant species, notably conifers that have historically been the primary source of turpentine oil, rich in alpha‐pinene and beta‐pinene [13, 14]. Among the various terpenes found in nature, citronellic acid, a monoterpene acid, deserves special attention for its biological properties, which are used in the cosmetic industry as a valuable ingredient of various products [15].

The dermis is the primary target for most anti‐wrinkle peptides. To be effective, these peptide molecules must penetrate the stratum corneum without being significantly degraded by proteolytic enzymes. Subsequently, these peptide molecules must reach the dermis at the appropriate concentrations to exert their anti‐wrinkle effects. Therefore, permeation through the viable epidermis is essential for the efficacy of most anti‐wrinkle peptides [16, 17].

Snake venom tripeptide is a hydrophilic tripeptide with good hydrophilicity but poor lipophilicity, which results in its limited ability to penetrate the stratum corneum, leading to low bioavailability. Therefore, it is necessary to modify and improve the chemical structure of snake venom tripeptide.

HY‐1 was a three‐peptide conjugate consisting of snake venom peptide and citronellic acid. We hypothesized that the terpene enhancement effects might increase the permeation of the conjugated systems as well, thereby demonstrating a novel skin‐penetrating peptides [18].

The goal of this study was to determine the in vitro effect and clinical skin efficacy of HY‐1. The ROS production rate model was used to determine how to scavenge ROS to confirm the effect of HY‐1 on skin aging function. The maintenance and soothing effects of HY‐1 were explored by detecting the cell migration rate and the relative expression of the IL‐6 gene. Finally, a split‐face clinical trial was conducted to assess the anti‐wrinkling effect of HY‐1 for 32 subjects with facial crow's feet and underfoot wrinkles (involved wrinkles grade ≥ 2).

## 2 Materials and Methods

### 2.1 Preparations of HY‐1

HY‐1 was prepared following a routine synthesis protocol of 7‐step organic reactions involving the use of typical reagents in the homogeneous synthesis of peptides, such as a base (lithium hydroxide), protecting groups(methyl), activating agents (N‐hydroxy succinimide), and solvents for reactions. All the desired intermediates were purified via column chromatography. The final product was subjected to chromatographic separation (Shim‐pack gist C‐18 column 5 μM, 4.6 × 150 nm; Shimadzu) and lyophilization process.

**FIGURE 1:**
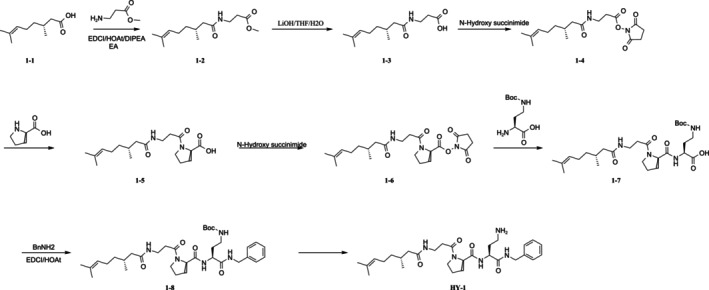
Synthesis process of HY‐1.

All chemical reagents were purchased from Adamas, Energy, and shanghai bide. The Synthesis process of HY‐1 was a conventional method for liquid phase chemical peptide synthetic methodology referred to a patent procedure with a little modification and shown in Figure 1 [19].

### 2.2 Characteristics of HY‐1

The molecular formula of HY‐1 was C_29_H_45_lN_5_O_4_, characterized by LC–MS, which found the MW peak [M + H_3_O]^+^: 546.7 (Agilent 1290+). HPLC was performed on a Shimadzu HPLC data system with P580 and UVD100 detectors (Shimadzu). The column temperature was 25°C, the flow rate was 1.0 mL/min, and the injected volume was 10 μL.

### 2.3 Cell Culture and Treatments

The human skin fibroblast (HSF) cell line was obtained from the Center for the Preservation of Chinese typical cultures at Wuhan University and cultured in DMEM medium supplemented with 10% heat‐inactivated FBS. HSF cells were used within about Tenth generations.

The human immortalized keratinocytes (HaCaT) cell line was obtained from the Center for the Preservation of Chinese typical cultures at Wuhan University and cultured in DMEM medium supplemented with 10% heat‐inactivated (fetal bovine serum, FBS). HaCaT cells were used within about 10 generations.

### 2.4 Measurement of ROS Generation

HSF cells were grown in 35 mm plates overnight. Then, the blank control cells were incubated in serum DMEM. The model control cells were incubated in serum DMEM with lipopolysaccharide (LPS, Sigma‐Aldrich). The sample cells were incubated in serum DMEM with various concentrations of HY‐1 and LPS. All the cells were incubated in a humidified atmosphere of 5% CO_2_ at 37°C for 24 h. After that, the medium was discarded, and the cells were washed three times with PBS. 1 mL DMEM (10%FBS, Sigma‐Aldrich) prepared with DCFH‐DA (1:1000, Sigma‐Aldrich) was added to each well. Then, the cells were placed into incubation at 37°C away from light for 30 min. At the end of the incubation, the cells were washed three times with PBS, and the ROS generation measurement was observed by photographing with fluorescence microscopy (DM18, DMi8, Leica).

### 2.5 RNA Extraction and Quantitative Real‐Time PCR (qPCR) Analysis

HaCaT cells in the logarithmic growth phase were seeded in cell culture dishes. When the cell fusion reached about 70%, the culture medium was discarded. The blank control cells and model control cells were replaced with fresh medium, and the sample cells were replaced with fresh medium containing various concentrations of HY‐1. Cells were cultured in an incubator at 37°C and 5% CO_2_ for 24 h. Except for the blank control group, the cells in the other groups were simultaneously irradiated with a UVB ultraviolet crosslinking apparatus for 40 s and then cultured in an incubator for 12 h. RNA was collected from all groups of cells, reverse transcribed into complementary DNA (cDNA), and the changes in the expression levels of the IL‐6 gene were quantified using a quantitative PCR instrument.

### 2.6 Measurement of the Effects on Nourishing, Firming, and Anti‐Wrinkle

The study to evaluate the effects on nourishing, firming, and anti‐wrinkle after using the product twice daily for 14 and 28 days included 32 participants with facial crow's feet and under‐eye wrinkles (with the involved wrinkle grades being ≥ 2), and ultimately 32 participants completed the study. The age range was 26–54 years with 1 male and 31 females, having an average age of 42.00 ± 7.75 years.

Patients’ inclusion criteria:

1. Chinese aged 20–55 who are in good health.
2. Physically healthy, without any other chronic diseases or ongoing treatments.
3. Voluntarily participate in the evaluation and sign an informed consent form.
4. Willing to comply with all evaluation requirements.

Patients’ exclusion criteria:

1. Those who have used antihistamines in the past week or have tried immunosuppressive drugs within the past month.
2. Those who have used any anti‐inflammatory drugs at the test site within the past 2 months.
3. The subject has clinically unhealed inflammatory skin disease.
4. Insulin dependent diabetes patients.
5. Patients with asthma or other chronic respiratory diseases are undergoing treatment.
6. Individuals who have received anticancer chemotherapy within the past 6 months.
7. Patients with immunodeficiency or autoimmune diseases.
8. Breastfeeding or pregnant women.
9. Patients who have undergone bilateral mastectomy and bilateral axillary lymph node dissections.
10. The test results are affected by scars, pigmentation, atrophy, erythematous nevi, uneven skin color, folliculitis, or other defects in the skin test site.
11. Participate in other clinical trial researchers.
12. Individuals with a highly sensitive physical constitution.
13. Non‐voluntary participants or those who are unable to complete the prescribed content according to the experimental requirements.

Instrument involved: CorneometerCM825 measures the moisture content of the stratum corneum, Cutometer dual MPA580 measures skin elasticity R2 value, skin firmness F4 value, and skin stretch height R0 value, Glossymeter GL200 measures skin glossiness, Ultrascan UC22 measures the thickness and density of the skin's dermis layer, and VISIA Image Capture.

Software involved: The Wilcoxon signed rank test was used as a statistical method, and the software SPSS was used.

## 3 Results

### 3.1 Effects of HY‐1 on ROS Production

**FIGURE 2:**
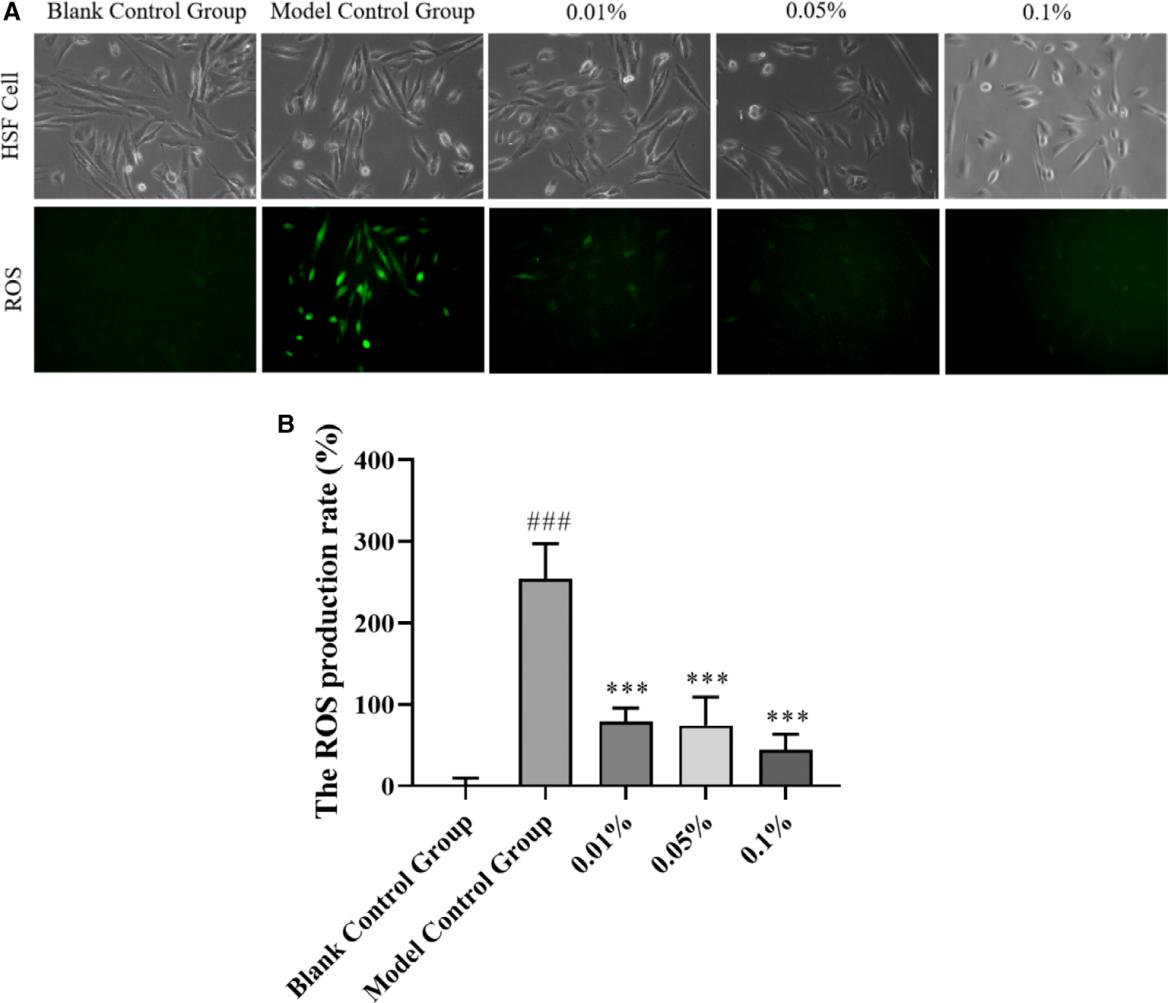
(A) Effect of HY‐1 on the ROS production rate in HSF cells measured by fluorescence. (B) ROS production rate in HSF cells measured by fluorescence.

The effects of 0.01%, 0.05%, and 0.1% HY‐1 on the ROS production rate in HSF cells are shown in Table 1 and Figure 2. The ROS production rate in the model control group significantly increased to 253.82% ± 17.67% compared to the blank control group (0.00% ± 4.19%) (*p* < 0.001), indicating that the oxidative stress model in HSF cells was successfully established. Under the same conditions, the ROS production rates in HSF cells treated with 0.01%, 0.05%, and 0.1% HY‐1 were 78.34% ± 7.05%, 74.27% ± 17.22%, and 44.43% ± 8.57%, respectively, which were significantly reduced by 175.48% (*p* < 0.001), 179.55% (*p* < 0.001), and 209.39% (*p* < 0.001) compared to the model control group. This indicates that HY‐1 has the ability to scavenge ROS (highly significant) under these experimental conditions.

**TABLE 1:** Effect of HY‐1 on the ROS production rate in HSF cells.

Group	Blank control	Model control	0.01%	0.05%	0.1%
ROS production rate	0.00 ± 4.19	253.82 ± 17.67	78.34 ± 7.05	74.27 ± 17.22	44.43 ± 8.57
*p*	—	< 0.001	< 0.001	< 0.001	< 0.001

The generation of ROS is one of the skin health damages caused by UV radiation. Oxidative stress and its relative oxidative damage can aggravate skin pigmentation and aging, leading to wrinkling, sagging, and roughness [20, 21]. In vitro experiments, ROS formation was significantly reduced in HSF cells pretreated with three different concentrations of HY‐1 compared to the model control group, as expected.

### 3.2 Effects of HY‐1 on Interleukin‐6 (IL‐6) mRNA Expression

**FIGURE 3:**
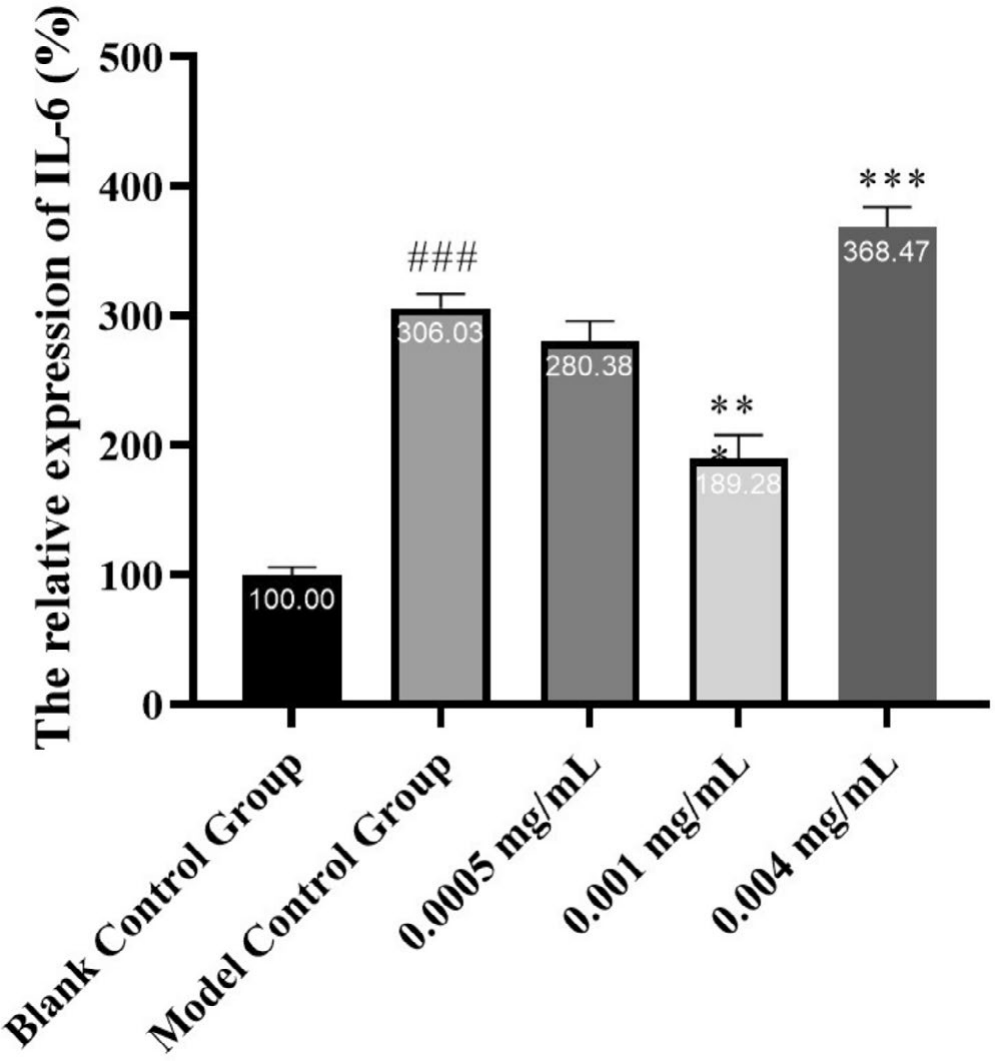
Relative expression levels of the IL‐6 gene measured by fluorescence. Notes: n.s. *P* > 0.05 versus baseline; *0.01 ≤ *P* < 0.05 versus baseline; **0.001 ≤ *P* < 0.01 versus baseline; *** *P* < 0.001 versus model control.

The relative expression levels of the IL‐6 gene after treatment with 0.0005, 0.001, and 0.004 mg/mL of HY‐1 were 280.38% ± 15.52%, 189.28% ± 18.56%, and 368.47% ± 15.61%, respectively, as shown in Figure 3. Compared to the model control group, the expression was downregulated by 25.65% (*p* > 0.05), 116.75% (*p* < 0.001), and upregulated by 62.44% (*p* < 0.001), respectively. The treatment with 0.001 mg/mL HY‐1 significantly inhibited the expression of the IL‐6 gene, indicating its very pronounced inhibitory effect. HY‐1 exhibits reparative and soothing effects, particularly at the concentration of 0.001 mg/mL, which significantly suppresses the expression of the IL‐6 gene. These findings suggest the potential of HY‐1 as a therapeutic agent for skin repair and soothing.

### 3.3 HY‐1 Improved Clinical Benefits on Nourishing, Firming, and Anti‐Wrinkle After 28 Days

**FIGURE 4:**
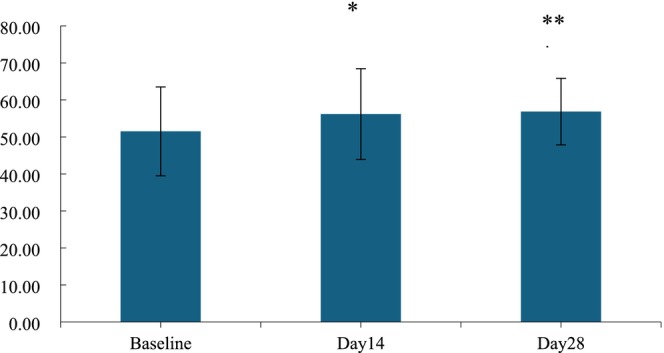
Value of skin elasticity R2 before and after use. n.s., *p* > 0.05 versus baseline; *0.01 ≤ *p* < 0.05 versus baseline; **0.001 ≤ *p* < 0.01 versus baseline, ***p < 0.001 versus baseline.

**FIGURE 5:**
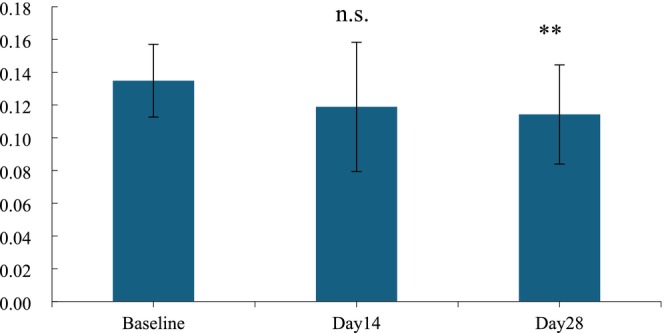
Value of skin stretch height R0 before and after use. n.s., *p* > 0.05 versus baseline; *0.01 ≤ *p* < 0.05 versus baseline; **0.001 ≤ *p* < 0.01 versus baseline, ***p < 0.001 versus baseline.

**FIGURE 6:**
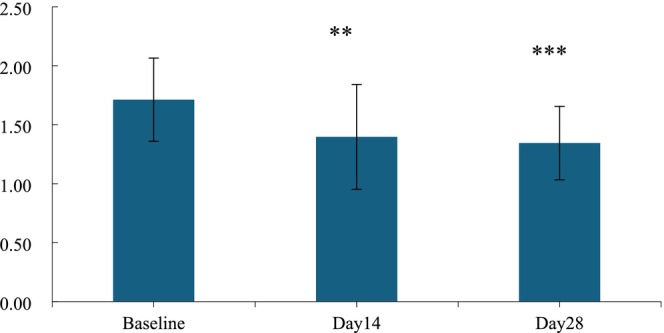
Value of skin firmness F4 before and after use. n.s., *p* > 0.05 versus baseline; *0.01 ≤ *p* < 0.05 versus baseline; **0.001 ≤ *p* < 0.01 versus baseline, ***p < 0.001 versus baseline.

The changes in skin firmness are characterized by three parameters: skin elasticity R2, skin firmness F4, and skin extensibility R0. Significant differences were observed and measured at Days 14 and 28. Compared to before use, after using the HY‐1 for 14 days, the skin elasticity R2 value, skin extensibility R0, and skin firmness F4 improved by 9.07%, 11.85%, and 18.41% (*p* < 0.05, Figures 4, 5, 6). Additionally, On the 28 days of treatment, the skin elasticity R2 value, skin extensibility R0, and skin firmness F4 further increased by 10.36%, 15.27%, and 21.49% (*p* < 0.05).

Summarizing the data on the 14th and 28th days, remarkable changes in skin firmness were observed, indicating that HY‐1 has an effect on tightening skin.

**FIGURE 7:**
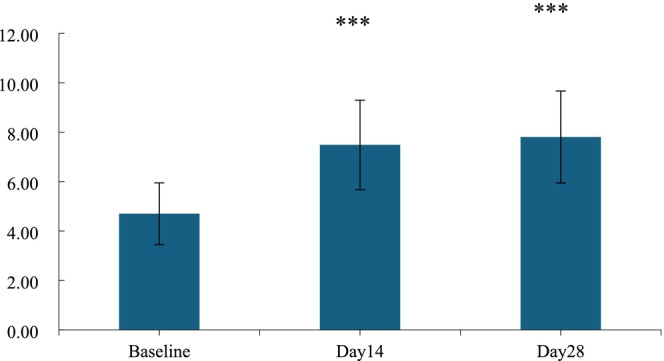
Value of skin gloss before and after use. n.s., *p* > 0.05 versus baseline; *0.01 ≤ *p* < 0.05 versus baseline; **0.001 ≤ *p* < 0.01 versus baseline; ***p < 0.001 versus baseline.

**FIGURE 8:**
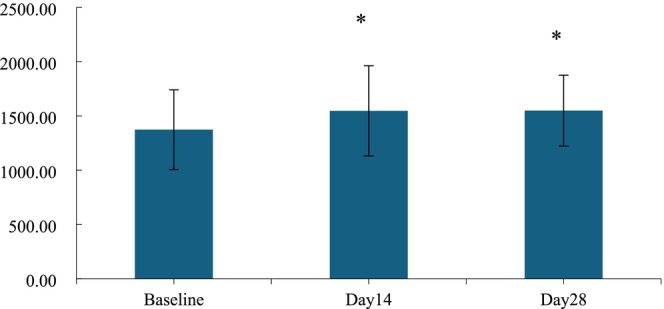
Thickness of the skin dermis before and after use. n.s., *p* > 0.05 versus baseline; *0.01 ≤ *p* < 0.05 versus baseline; **0.001 ≤ *p* < 0.01 versus baseline; ***p < 0.001 versus baseline.

There were two parameters: skin glossiness and the density of the skin's dermis layer reflecting the changes in skin nourishment. After a 14‐day application of the HY‐1, there was a 59.23% enhancement in skin glossiness, marking a noticeable improvement. However, when assessing the skin's dermis layer thickness after a 14‐day application, the increase of 12.62% did not reach statistical significance. Upon using the HY‐1 for 28 days, the dermis layer of the skin showed an increase in thickness of 12.79%, and this change was found to be statistically significant, as shown in Figures 7 and 8.

**FIGURE 9:**
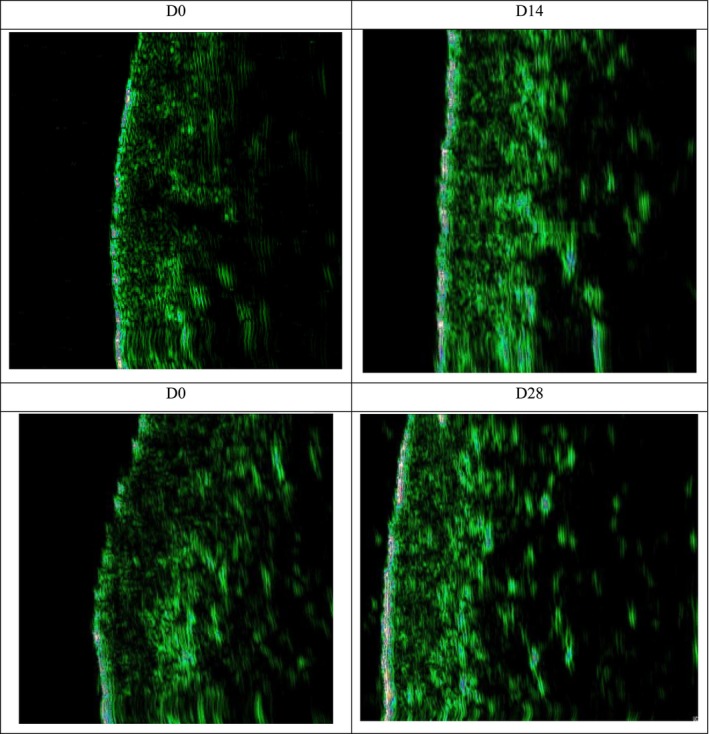
Graph of the thickness of the skin dermis before and after 14 and 28 days.

As additional evidence of skin nourishment improvement, the thickness of the dermis layer was also detected, as shown in Figure 9. The results were consistent with the above two parameters. After 14 and 28 days of treatment with HY‐1, the increase rates of skin dermis thickness were 12.62% and 12.79%, respectively.

Taking into account the three parameters, skin elasticity R2, skin glossiness, and skin dermis density, all have shown significant improvement in the skin situation, indicating that HY‐1 has nourishing effects.

### 3.4 HY‐1 Improves Clinical Benefits on Wrinkles After 28 Days

Crow's feet and under‐eye wrinkles are the primary facial wrinkles under examination, with a focus on detecting their depth and area.

**FIGURE 10:**
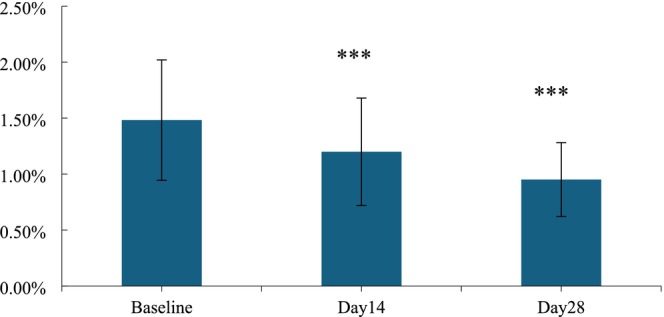
Area ratio of crow's feet before and after use. n.s., *p* > 0.05 versus baseline; *0.01 ≤ *p* < 0.05 versus baseline; **0.001 ≤ *p* < 0.01 versus baseline; ***p < 0.001 versus baseline.

**FIGURE 11:**
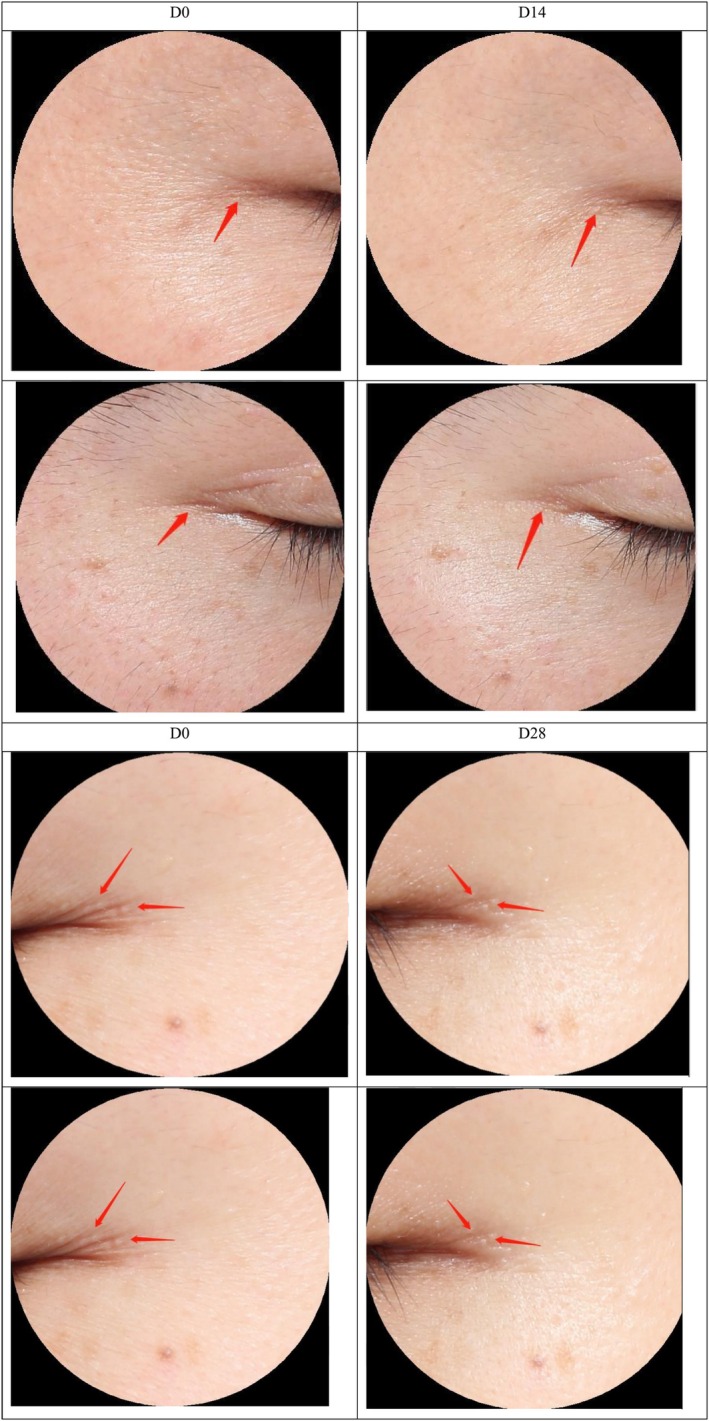
Photo of the patients' crow's feet before and after use 14 and 28 days.

Post‐application of the HY‐1, there was a notable improvement in the area of crow's feet by 19.08% after 14 days treatment, indicating a significant improvement. Similarly, after a 28‐day application period, the area of crow's feet demonstrated a more pronounced improvement, with the rate escalating to 35.84%, marking a significant difference from the initial state. (*p* < 0.001; Figures 10 and 11).

**FIGURE 12:**
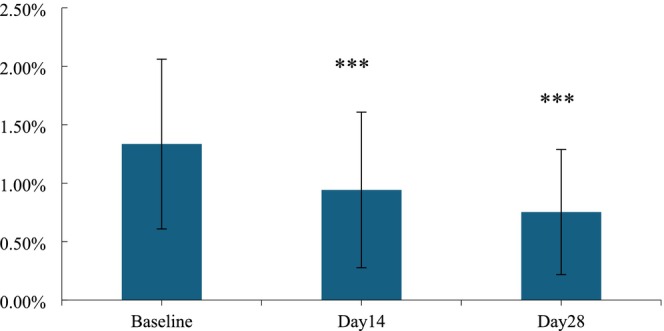
Area ratio of under‐eye wrinkles before and after 14 and 28 days of use. n.s., *p* > 0.05 versus baseline; *0.01 ≤ *p* < 0.05 versus baseline; **0.001 ≤ *p* < 0.01 versus baseline; ***p < 0.001 versus baseline.

**FIGURE 13:**
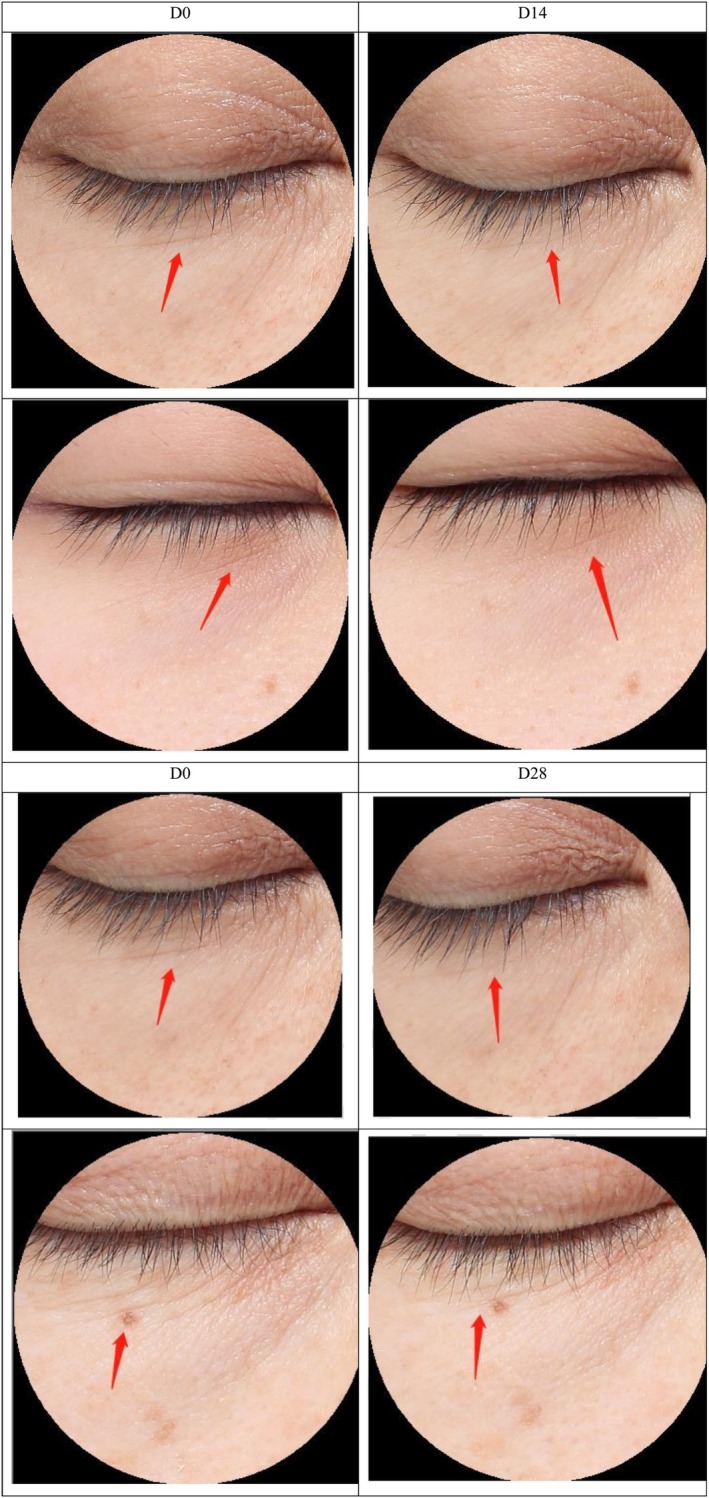
Photo of the patients' under‐eye wrinkles before and after 14 and 28 days of use for 14 and 28 days.

As an additional criterion for measuring anti‐wrinkle effects, the under‐eye wrinkles were also improved after treatment with HY‐1. In comparison to the baseline measurements, a significant improvement of 29.44% was observed in the area percentage of under‐eye wrinkles after a 14‐day application of the HY‐1 (*p* < 0.001). Upon extending the application period to 28 days, the improvement rate further increased to 43.60%, which presented a significant reduction in the area percentage of under‐eye wrinkles when compared to the pre‐application state (*p* < 0.001; Figures 12 and 13).

The study findings corroborate the anti‐wrinkle efficacy of HY‐1. The HY‐1 demonstrated a capacity to significantly reduce the area percentage of both under‐eye wrinkles and crow's feet without eliciting adverse skin reactions, thereby validating its potential as an effective antiaging cosmetic intervention.

## 4 Discussion

HY‐1 is a three‐peptide derivative from Syn‐Ake, a tripeptide marketed by pentapharm, with a mode of action similar to that of Waglerin 1, that has demonstrated its capacity to reduce wrinkles through the inhibition of muscle contractions [22]. The objective of the current work was to show HY‐1 as an alternative and effective topical ingredient for combating visible signs of aging without the side effects such as redness or irritation.

### 4.1 In Vitro Effects of HY‐1 on ROS‐Scavenging

Skin serves as the primary interface between our body and the external environment and is subjected to oxidative stress caused by various factors, including solar ultraviolet, environmental pollution such as ozone and particulate matter, and psychological stress. Excessive reactive species, such as ROS. exacerbate skin pigmentation and aging, leading to issues such as uneven skin tone, pigmentary disorders, skin roughness, and wrinkles [23].

ROS at low concentrations exert their physiological activity, but excessive ROS levels are harmful and can cause severe cellular damage. The unique reticular structure of collagen maintains skin elasticity and tension. ROS disrupts fibroblast biosynthesis, including collagen, which leads to skin relaxation [23, 24]. In vitro settings, HSF was cultured in LPS and LPS + HY‐1 for 24 h, respectively. ROS formation was significantly reduced in the HY‐1–treated group compared to the vehicle, demonstrating a potential anti‐wrinkle effect in clinical.

### 4.2 In Vitro Effects of HY‐1 on Anti‐Inflammatory and Soothing Effects

The secretion of proinflammatory cytokines serves as an early biomarker for skin damage and UV‐irritation [25]. IL‐6 is a cytokine associated with inflammation and aging, and an elevated expression level of IL‐6 is a notable characteristic of the inflammatory response and skin aging [26, 27]. Therefore, by monitoring the changes in IL‐6 expression, we can investigate whether HY‐1 can inhibit the expression of IL‐6, thereby possessing anti‐inflammatory effects.

The analysis of IL‐6 expression showed that 0.001 mg/mL HY‐1 did not trigger a primary inflammatory response and showed anti‐inflammatory properties in vitro. This finding validates HY‐1 as an intriguing anti‐inflammaging skincare component.

### 4.3 Clinical Effects of HY‐1

Our results, obtained by ROS and the thickness of skin dermis tests, showed that HY‐1 may work on anti‐wrinkles through various mechanisms like antioxidation and regeneration of collagen. These findings were consistent with the clinical trials. The data from IL‐6, skin nourishing, and firming tests also showed that HY‐1 has multiple effects. Therefore, treatment with HY‐1 may solve different skin problems at the same time.

We acknowledge several limitations in our study. It is important to consider seasonal factors in the evaluation of anti‐winkles. Our study was performed in summer and thus did not include autumn and winter. The study was controlled, and the subjects were sequentially allocated to the treated group. The placebo and reference groups should be led into future trials. More mechanistic research should be carried out to investigate the new ingredient.

## 5 Conclusion

Taken together, the results from in vitro and clinical studies indicate that HY‐1 offers multiple skin benefits by smoothing the skin, with anti‐inflammatory and anti‐wrinkle effects.

HY‐1 serves as a viable skincare alternative targeting the visible signs of aging and photodamage associated with currently available technologies. This novel and multifunctional ingredient achieves notable clinical benefits in skin firmness and smoothing by enhancing both epidermal and dermal parameters, with no evidence of erythema or irritation observed up to 28 days of continuous use. This new ingredient could represent a good option for the management of wrinkles. Furthermore, IL‐6 gene expression results suggest that HY‐1 has anti‐inflammatory effects, while increased epidermal thickness indicates skin renewal properties.

Nonetheless, further research is essential to substantiate the efficacy and safety of HY‐1 for extended use beyond the initial 28‐day period, ensuring the maintenance of its beneficial effects without inducing side effects.

## Author Contributions

Xilei Duan: investigation; methodology; writing – original draft. Zhong Lu: conceptualization; data curation; methodology; project administration; supervision; writing – review and editing.

## Conflicts of Interest

The authors declare no conflicts of interest.

## Data Availability

Research data are not shared.
